# Distribution and hormonal regulation of membrane progesterone receptors β and γ in ciliated epithelial cells of mouse and human fallopian tubes

**DOI:** 10.1186/1477-7827-7-89

**Published:** 2009-08-28

**Authors:** Magdalena Nutu, Birgitta Weijdegård, Peter Thomas, Ann Thurin-Kjellberg, Håkan Billig, DG Joakim Larsson

**Affiliations:** 1 Department of Neurosciences and Physiology, the Sahlgrenska Academy, University of Gothenburg, Box 434, SE-40530, Gothenburg, Sweden; 2 Department of Obstretics and Gynecology, Sahlgrenska University Hospital, the Sahlgrenska Academy, University of Gothenburg, Blå Stråket 6, SE-41345, Gothenburg, Sweden; 3 Marine Science Institute, University of Texas at Austin, 750 Channel View Drive, Port Aransas, TX 78373, USA

## Abstract

**Background:**

The controlled beating of cilia of the fallopian tube plays an important role in facilitating the meeting of gametes and subsequently transporting the fertilized egg to its implantation site. Rapid effects of progesterone on ciliary beat frequency have been reported in the fallopian tubes of cows, but the identity of the receptors mediating this non-genomic action of progesterone is not known. We recently identified a member of the non-genomic membrane progesterone receptor family, mPR gamma, as a candidate for mediating these actions of progesterone. Here, we investigated the possible presence of a related receptor, mPR beta, in the fallopian tubes of mice and women as well as the possible hormonal regulation of mPR beta and gamma.

**Methods:**

Western blot and immunohistochemistry with specific antibodies were used to characterize the expression and cellular localization of the mPRs in mouse and human tissues. Taqman (Quantitative Polymerase Chain Reaction) assays were used to quantify mRNA levels in the fallopian tubes of two different mouse models after injections with different hormones and specific antagonists.

**Results:**

In the fallopian tubes of both mouse and human, the expression of mPR beta and mPR gamma proteins was exclusively found in the ciliated cells. Whereas mPR beta was found on the cilia, mPR gamma was localized at the base of the same ciliated cells, as previously reported. In gonadotropin-primed mice, both mPRs genes were down-regulated after an injection with progesterone. Treatment with estradiol rapidly down-regulated the level of mPR beta mRNA and protein in immature mice. The mPR gamma protein was down-regulated around the time of ovulation in cycling women, similar to the regulation observed in mice stimulated to ovulate via gonadotropin injections.

**Conclusion:**

Our findings show the presence and hormonal regulation of two distinct mPRs associated with the cilia of the fallopian tubes in both mice and women. It is hypothesized that these receptors are involved in the control of ciliary movement and, thus, gamete transport in the fallopian tubes of mammals.

## Background

The fallopian tube (oviduct) of mammals consists mainly of muscular, ciliated and secretory cells. A central function of the fallopian tube is to facilitate gamete transport, fertilization and early embryonic development [1]. Gamete transport in the fallopian tube is reported to depend on both smooth muscle contractility and the action of cilia [2]. However, inhibition of muscle contractility by isoproterenol did not affect ovum transport in fallopian tube of rats [3], and much of the literature supports ciliary activity as the most important factor regulating gamete transport rate [4,5]. Sex steroids are involved in the control of gamete transport and ciliary activity in the fallopian tube. Estradiol (E_2_) treatment accelerates ovum transport from 72 h to 24 hours in rats [6], facilitates sperm migration and induces adhesion of spermatozoa to the oviductal epithelium [7]. Concomitant treatment with progesterone (P_4_) blocked E_2_-induced ovum transport [8], whereas P_4 _alone retarded the process [8-11]. Progesterone also acts directly on the sperm membrane to promote sperm motility [12,13] and induces the acrosome reaction [14]. The cumulus cells surrounding the travelling ovum is a source for P_4 _in humans [15,16], rabbit [17], mouse [18] and pig [19]. It is possible that P_4 _secreted from the oocyte cumulus complex acts as a beacon to the cilia of the fallopian tube, indicating its detailed position [20]. Indeed, understanding the underlying mechanism of E_2 _and P_4 _action in the regulation of tubal function is important.

Traditionally, P_4 _is thought to mediate its effects through its nuclear receptors, PGR-A and PGR-B, which are often co-expressed within the same cell [21,22], to alter gene transcription. Not all effects of P_4_, however, can be explained by this classical mechanism of steroid action. It has been suggested that P_4 _together with PGR present in the cytoplasm could initiate a non-genomic signaling cascade [23]. Rapid responses to P_4 _have also been reported in tissues or cells naturally lacking nuclear PGR [24,25] and in *Pgr *knockout models [26], suggesting an involvement of other non-related receptors. [27]. A few years ago, a novel family of putative receptors (mPRs), including mPRα, β and γ, were identified [28,29]. The mPRs belong to the Class II progestin and adipoQ receptors family (PAQR) [30,31]. There is a growing set of data, to suggest a supporting role of the mPRs as functional progestin receptors in different vertebrates, although there are still some controversies about their function [32-35]. The best described forms are the mPRα and mPRβ proteins [33,35-42], whereas mPRγ is less studied [20,28,30].

We have previously shown that mPRγ is expressed at the apical cell membrane of ciliated cells in the mouse and human fallopian tube, leading us to hypothesize that mPRγ can be a mediator of rapid effects of P_4 _on ciliary activity in mammals [20]. Monkkonen et al. (2007) reported the expression of the mPRβ gene in a fallopian tube cell line possessing secretory epithelial cell properties [39], and Romero-Sanchéz et al. (2008) reported expression of the mPRβ and α protein in all epithelial cells of the human fallopian tube, but the intracellular localization of the receptors remained unclear [43]. In this study we demonstrate a distinct membrane-associated localization of both mPRβ and γ in ciliated cells of the fallopian tube in mice and women. Whereas mPRγ is found at the base of the cilia, mPRβ is located along the cilia themselves. Furthermore, we show regulation of both mPRβ and γ by sex steroid treatment in mice and a variable mPRβ expression in fallopian tubes of women during the menstrual cycle. Our results suggest the possibility for a cooperative role of mPRβ and γ in gamete transport.

## Methods

### Antibodies, hormones and reagents

For Western blot analysis and immunohistochemistry studies, the following primary and secondary antibodies were used: mPRβ [44], mPRγ [20], β tubulin-IV (T7941; Sigma-Aldrich, St Louis, MO), PGR (sc-539; C-20; Santa Cruz Biotechnologies Inc, Santa Cruz, CA) and FSHr (sc-7798; N-20; Santa Cruz Biotechnologies Inc). The alkaline phosphatase anti-rabbit IgG (T2191; Tropix, Bedford, MA), alkaline phosphatase anti-goat IgG (SC-2022; Santa Cruz Biotechnologies Inc), TexasRed anti-rabbit IgG (TI-1000, Vector lab Inc., Burligame, CA) and TexasRed anti-mouse IgG (TI 2020; Vector lab Inc.) were used as secondary antibodies.

Progesterone (P_4_) and 17-β-estradiol (E_2_) were from Sigma; ICI 182,780 (Faslodex™, AstraZeneca) was purchased from Tocris Cookson Ltd. (Bristol, UK), and CDB2914 was from Organon (N.V. Organon, Oss, and Netherlands). Equine gonadotropin (eCG; cat.no.G4877) was from Sigma-Aldrich (Saint Louis, MO), and human chorionic gonadotropin (hCG) was obtained from N.V. Organon. The protease inhibitor cocktail was from Roche Diagnostics (Roche Complete, Mannheim, Germany), and CDP-Star ready-to-use developing system was from Tropix. All chemicals used in immunohistochemistry or immunofluorescent staining, included in kits or alone, were purchased from Vector Laboratories, Inc., unless otherwise specified.

### Animal studies

All experiments were performed with the approval of the annual local ethics committee in Gothenburg, Sweden (246/2007 to D.G.J. Larsson). The animals, 352 females and three males C57BL/6 mice, used in these experiments were obtained from Taconic M&B, Copenhagen, Denmark.

### Experimental design and tissue preparation in mice

#### Tissue distribution of mPRβ in male and female mice

To examine the general tissue distribution of mPRβ, different organs from immature (21-day-old) male and female mice (n = 3 biological replicates per sex), were collected. Fat or connective tissues were immediately removed from the tissues, and the samples were directly frozen in liquid nitrogen for protein preparation and Western blot analysis (see below for details).

#### Localization of mPRβ and γ proteins in the mouse fallopian tube

To investigate the localization of mPRs in the fallopian tube, tissues from immature female mice (21-day-old) were collected and fixed in 10% neutral buffered formalin for immunohistochemistry analysis.

#### Regulation of mPRβ and γ in the fallopian tube of immature female mouse following hormonal treatment

The concentrations of E_2 _and P_4 _are very low in the immature mouse, allowing us to assess the effects of exogenous steroid addition with limited interference by endogenous steroids. To examine whether exogenous E_2 _and P_4 _regulate mPRβ and γ expression, individual groups of 21-day-old female mice were therefore given one i.p. injection E_2 _(0.3 mg/kg) or P_4 _(4 mg/kg). To investigate whether the nuclear estrogen receptors (ESRs) and PGRs are involved in the regulation of mPR, we used the ESR antagonist ICI 182,780 (ICI, 8.3 mg/kg) and the PGR-A/B antagonist CDB2914 (CDB, 1 mg/animal). All compounds were suspended in 100 μl of sesame oil (Sigma). After 2 h and 6 h of treatment, one group of animals (n = 5) was killed, and the tissues were frozen immediately for RNA preparation. To study the regulation of the mPR proteins, fallopian tubes were sampled 6 h and 24 h after injection with either E_2 _or oil and were frozen in liquid nitrogen for Western blot analysis. Whereas RNA analyses were performed on individual samples, pooling of tissue from ten individual mice was required for each protein analysis. Thus, the experiment with E_2 _and oil was repeated several times to generate three independent pools of tissue from each treatment group for the analyses of mPRβ and γ.

#### Regulation of mPRβ and γ in eCG-stimulated female mouse fallopian tube following hormonal treatment

In the mouse, the levels of expression of PGR and ESR are increased after hormonal treatment [45-47]. Therefore, to verify the effect of steroid treatment in a model expected to have higher levels of nuclear receptors, as well as being closer to a developmental stage when the control of gamete transport becomes important, we also used a gonadotropin-primed mouse model. Immature female mice (21-day-old) were given a single i.p. injection of 5 IU equine Chorionic Gonadotropin (eCG). After 48 h, the mice were injected i.p. with the steroid/antagonist and were sampled after 2 h or 6 h, as described under experiment three.

### Effects of gonadotropins on mPRβ and γ expression in mouse fallopian tube

To study the regulation of mPRβ and γ before and after ovulation and to allow a comparison with human data (see below), we used a mouse model where eCG was administered as in experiment four. After 48 h, mice were injected i.p. with 5 IU of human Chorionic Gonadotropin (hCG). In mice given this treatment, serum E_2 _concentrations are increased by 24 h after eCG administration, reaching a peak after 48 h, whereas P_4 _concentration are increased after post-hCG administration and peak around 48 h [46]. The serum levels of E_2 _and P_4 _in immature mice upon treatment with eCG/hCG is similar to those of adult cyclic mice [48]. At times 0, 48, 72 and 96 h from the onset of the first injection, animals were killed by cervical dislocation, and their fallopian tubes were dissected and frozen in liquid nitrogen for Western blot analysis.

### Human studies

Fallopian tube samples were obtained from 12 fertile women, aged 28-42 y, undergoing tubal ligations at the Sahlgrenska University Hospital. Prior to the operation, informed consent from the patients and approval by the institutional committee on the use of human subjects in Gothenburg (RS175-99 to A. Thurin) were obtained. All women had a clinical examination before surgery and had regular menstrual cycles (cycle length, 25-32 days). No woman had used hormonal medication within three months of surgery. The menstrual phase of each patient at the day of operation was classified based on previous menstrual history, serum steroids and luteinizing hormone-levels in urine [49]. A cross-section of the isthmus part of the fallopian tube was taken from each patient. One part was fixed with 10% neutral buffered formalin, embedded in paraffin, sectioned and mounted. The remainder was placed into cold saline solution prior to freezing (-70°C) for later Western blot analyses.

### RNA isolation and reverse transcription-polymerase chain reaction

Total cellular RNA was isolated from the fallopian tubes of individual mice using the RNeasy Micro Kit (Qiagen, Germany) according to the manufacturer's instructions and was treated with an RNase inhibitor (Applied Biosystems, Foster City, CA). A single batch of 0.5 μg of total RNA from each sample was used to synthesize single-stranded cDNA using High-Capacity cDNA Reverse Transcription Kits (Applied Biosystems). Primers targeting the cDNA sequence of mPRβ (sense, 5'-CGGCGGCTGCTTTCTGT-3' and antisense, 5'-TAAGGCCGTCGGTAGCGATA-'3), mPRγ (sense, 5'-CTCCCTAGGCTATTCCGCATAG-3' and antisense, 5'-GGATGCCCTGCTCATGGA-3') and GAPDH, used as an endogenous control (sense, 5'-TGTGTCCGTCGTGGATCTGA-3' and antisense, 5'-ATGCCTGCTTCACCACCTTCT-3') were designed by using Primer Express (Applied Biosystems) and purchased from Cybergene AB, Stockholm, Sweden. The corresponding cDNA fragments were denatured at 98°C for 2 min, annealed at 59°C for 30 sec. The number of RT-PCR cycles was chosen to generate detectable signals without approaching saturation in any samples. After 30 cycles of amplification, the PCR products were isolated by electrophoresis and visualized using ethidium bromide.

### Taqman quantitative polymerase chain reaction

The Taqman QPCR reaction (quantitative polymerase chain reaction) was performed with the ABI Prism 7000 Sequence Detector (Applied Biosystems). The dissociation graph showed only one specific amplicon product for each target. For each amplification reaction, 20 ng cDNA, 500 nM of each primer and 1 × Sybr^®^Green PCR reaction mix (Applied Biosystems) was combined into a final volume of 25 μl. The PCR parameters were set according to the manufacturer's protocol. All reactions were performed in duplicate for both target genes and endogenous controls. The efficiencies of target and the endogenous controls were similar. Because not all samples could be run at the same time, one standard sample was run five times as duplicates in all assays to compensate for possible run to run variations. The expression of each target gene was normalized to the endogenous control (GAPDH) by subtracting the CT- value from the target gene with the CT-value of the endogenous control. To compare levels relative to a calibrator (mean of Group 1), ΔCT of group 1 was subtracted from the ΔCT of each sample. Relative expression is given by 2^-ΔΔCt^.

### Protein extracts and Western blot analysis

All protein preparations of fallopian tube samples were performed as described by Fujii et al., (2001) [50]. Samples from male and female mice used in the tissue distribution experiment were prepared according to Bordier et al. (1981) and Nutu et al., (2007). Thirty μg of protein were run by electrophoresis on 4-12% one-dimensional Bis-Tris gels (Novex, San Diego, CA, USA). In experiments in which both mPRβ (1:5000), mPRγ (1:100), PGR (1:250) and follicle stimulating hormone receptor (FSHr; 1:250) were assessed, the gels were cut into two pieces along a line corresponding to approximately 60 kDa. The section containing higher molecular weights was exposed to antibodies against PGR, or FSHr, and the lower molecular weight section was exposed to antibodies against mPRβ or γ. Separate gels and membranes were run for the two mPRs as they have very similar molecular masses. The immunosignal-CDP-Star ready-to-use substrate for the alkaline phosphatase system (Tropix, Bedford, MA, USA) was used to visualize protein bands. Immunoblotted signals were quantified by densitometry using the Quantity One Software package (version 4.2, BioRad, Hercules, CA) directly after detection in a Fluor-S multi-imager (Bio-Rad Laboratories, Sundbyberg, Sweden). The loading was evaluated by staining the gels with Coomassie blue. Signal intensities of the individual protein were normalized to the gels stained with Coomassie blue and presented as ratios that represent arbitrary densitometric units (ADU) of relative abundance.

### Immunohistochemical analysis and confocal microscopy

Single and dual-fluorescence immunohistochemistry were based on the previously described methodology [20]. Approximately 4 μm thick tissue sections were placed on glass slides, deparaffinized, rinsed in ethanol and re-hydrated through a series of decreasing concentrations of ethanol. Antigens were retrieved by boiling with an antigen unmasking solution for 20 min at 120°C, which was followed by cooling in dH_2_O for 5 min. Nonspecific binding was blocked with the background sniper (Biocare medical, Concord, CA USA), for 10 min at a room temperature. The sections were incubated in a humidified chamber at 4°C over night, with either a polyclonal antibody against mPRβ at dilution of 1:600 or a mouse monoclonal anti-β-tubulin IV mouse at a dilution of 1:600 in TBS. The MACH 3 rabbit AP (alkaline phosphatase) polymer kit and MACH 3 mouse HRP (horse radish peroxidase) polymer kit (Biocare Medical) were used as the detection systems, and the immunostaining was visualized by using Vulcan Fast Red Chromagen Kit and Romulin AEC Chromagen Kit (Biocare Medical) according to the manufacturer's protocol. Sections were counter stained with hematoxylin, dehydrated through a series of increasing concentrations of ethanol to clear the tissue and cover-slipped. The immunostaining and detection of mPRγ protein were performed using the same protocol as previously reported [20]. Negative control slides were prepared in an identical manner and processed with a ready-to-use mouse or rabbit IgG negative control (Biocare Medical) instead of the primary antibody. Slides were viewed on a Nikon EFD-3 (Nikon, Tokyo, Japan) microscope under bright field optics and photographed. The immunofluorescences on the slides were viewed on an Axiovert 200 confocal microscope (Carl Zeiss, Jena, Germany) equipped with a laser-scanning confocal imaging LSM 510 META system (Carl Zeiss) and was photomicrographed. Background settings were adjusted from examination of negative control specimens. Images of positive staining were adjusted to make the optimal use of the dynamic range of detection. Figures were composed in Adobe Photoshop with minimal alteration for presentation and layout.

### Statistical analysis

Data were analyzed using Minitab^® ^Statistical Software (Minitab Ltd., Coventry, UK). Differences between group means were analyzed using the general linear model (GLM). Post-hoc tests was applied were appropriate, including Dunnet's test for multiple comparisons to a control group or Bonferroni's test for comparisons between all groups. Residual analyses were used to check assumptions of normality and equality of variances and log-transformed when appropriate. Data are presented as mean ± SEM. P-values < 0.05 were considered statistically significant.

## Results

### Tissue distribution of mPRβ in male and female mice

**Figure 1:**
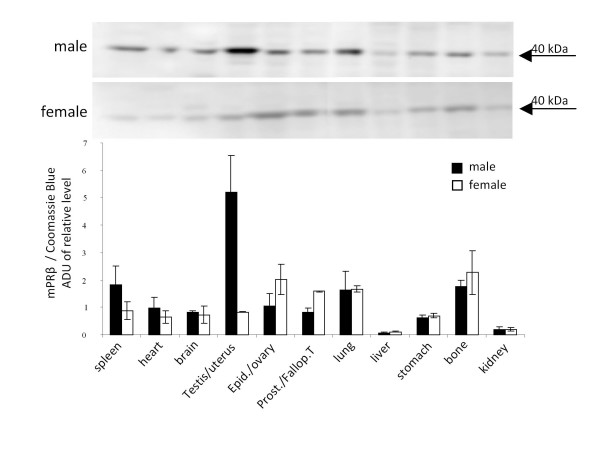
**Western blot analysis of mPRβ in tissues of male and female mice**. Densitometric quantification is indicated by the bar graph. Data are presented as mean arbitrary density units normalized to total protein loading ± SEM from three biological replicates per tissue.

We previously reported the tissue distribution of mPRγ in male and female mice [20]. [46]To determine the distribution of mPRβ protein in the same model, Western blot analysis was performed using a well-characterized antibody. The expression of mPRβ protein was confirmed by a single band of 40 kDa in female reproductive tissues (ovary, fallopian tube and uterus). High expression of mPRβ was also detected in male testis, whereas lower levels were observed in other tissues of both sexes (Fig. 1). The liver and kidney of both male and female mice showed the lowest, but still detectable, levels of mPRβ.

### Localization of mPRβ and mPRγ proteins in the mouse fallopian tube

**Figure 2:**
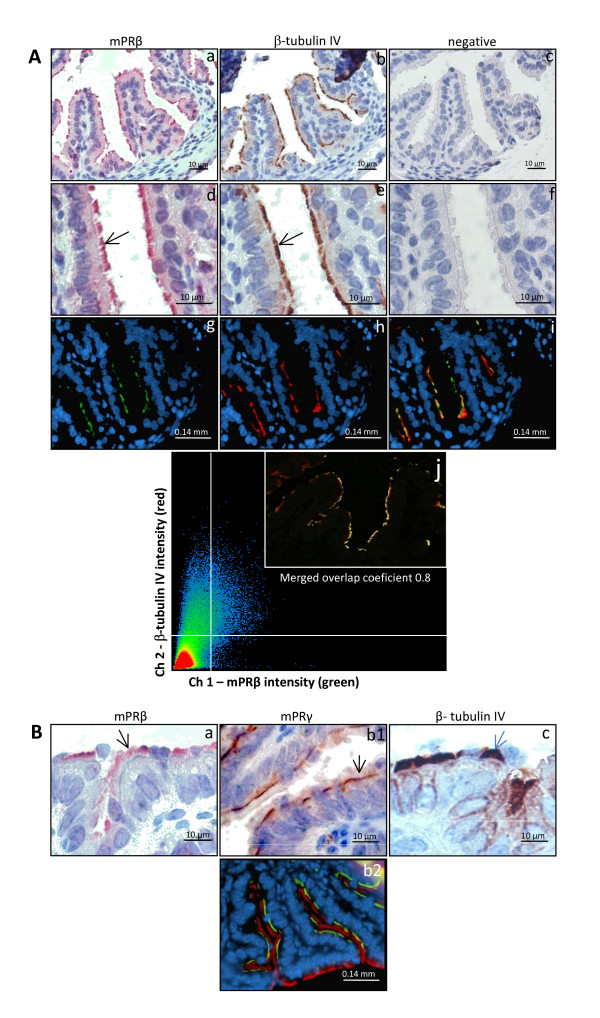
**Immunostaining for mPRβ, mPRγ and β-tubulin proteins in the mouse fallopian tube**. **A**: Immunohistochemical (**a - f**) and immunofluorescent (**g - j**) detection of mPRβ and β-tubulin, a marker for ciliated epithelial cells. Sections immunolabeled for mPRβ were visualized with MACH 3 rabbit AP (**a **and **d**) and green florescence (**g**). The β-tubulin was stained with MACH 3 mouse HRP (**b **and **e**) and red fluorescence (**h**). The panels' **c **and **f **are control sections where the primary antibody was omitted. A double staining was also performed using green and red fluorescent labels for mPRβ and β-tubulin, respectively (**i**). All fluorescence sections were subsequently counterstained with DAPI to visualized cell nuclei. Confocal microscopy in (**j**) is showing the overlapping fluorescence of two fluorochromes used. Tubal sections were labelled for mPRβ (green), β-tubulin (red) and merged (yellow). **2B**: To facilitate a comparison of the localization of the mPRs, immunostaining for mPRβ (**a**) mPRγ (**b1**) β-tubulin (**c**) and dual staining of mPRγ (green) and β-tubulin (red) (**b2**) are also presented.

The mPRβ protein was expressed in the fallopian tube of immature female mice (Fig. 2A). The localization was confirmed by immunohistochemistry (2A.a and d) and immunofluorescence (2A. g, i and j). The mPRβ was only found in the cilia of epithelial cells facing the lumen of the fallopian tube (c, d, f, and g). The identity of the cell type expressing mPRβ was verified using β-tubulin IV, a specific marker for ciliated cells (2A. b, e and h). Confocal microscopy also showed co-localization of mPRβ (green) and β-tubulin (red) immunoreactivity (2A. i and j). The localization of mPRβ was restricted to the upper part of the epithelial cell cilia (2B.a), whereas mPRγ was associated with the apical membrane of the cells (2B. b1) as previously reported [20]. These results were also confirmed using immunohistochemistry (2B.b1) and dual immunofluorescence (2B.b2). Labeling with an antibody against β-tubulin IV showed a strong staining restricted to the cilia of epithelial cells (2B.c).

### Regulation of mPRβ and mPRγ in the fallopian tube of immature female mouse following hormonal treatment

**Figure 3:**
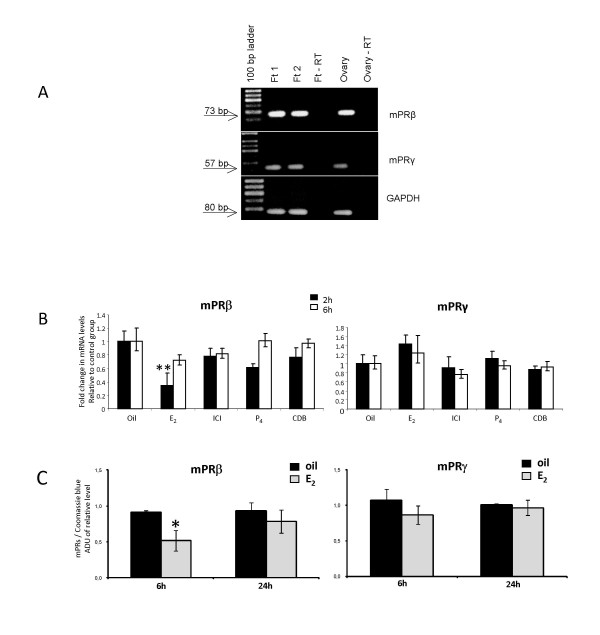
**Steroid-dependent regulation of mPRβ mRNA and mPRγ mRNA (A and B) and protein (C) in the fallopian tube of immature female mice**. Mouse ovary was included as a positive control in (**A**). Twenty-one-day-old female mice received an injection of E_2_, P_4_, the estrogen antagonist ICI 182,780, the progesterone antagonist CDB2914 or oil and sampled two or six hours later. The levels of mRNA are normalized to the endogenous control (GAPDH) and expressed in relative numbers to the control group as means ± SEM of five independent observations (**B**) whereas protein levels are presented as mean ± SEM of three independent pools, each consisting of tissue from ten mice (**C**). Groups differing significantly from the control group are indicated by asterisks (** for P < 0.01 and * for P < 0.05)

Considering that fallopian tubes from multiple mice are required for each Western blot analysis of mPR protein, we decided to first study the regulation of receptors on the mRNA level. As indicated in Fig. 3A, RT-PCR confirmed the expression of both mPRβ mRNA and mPRγ mRNA in immature mice.

Exposure to E_2 _rapidly and significantly decreased the mRNA expression of mPRβ but not of mPRγ, which had an opposite trend (Fig. 3B). The decrease in mPRβ mRNA was reflected in a similar decrease in protein levels (Fig. 3C). There were no significant effects of P_4_, ICI or CDB on either mPRβ and mPRγ gene expression two or six hours post injection (Fig. 3B). The standard deviations (SD) between the run to run samples were 0.008 cycles for the 2 h samples and 0.045 cycles for the 6 h samples and did not significantly affect the results; thus, no run-to-run compensation was included in the final calculations.

### Regulation of mPRβ and mPRγ in eCG-stimulated female mouse fallopian tube following hormonal treatment

**Figure 4:**
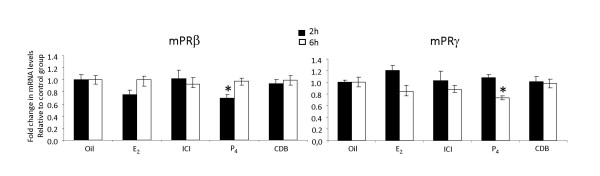
**Steroid-dependent regulation of mPRβ and γ in fallopian tubes of eCG-primed, immature mice**. Forty-eight hours after eCG injection, the mice received an injection of E_2_, P_4_, the estrogen antagonist ICI 182.780, the progesterone antagonist CDB2914 or oil and were sampled two or six hours later. The levels of the target genes are normalized to the endogenous controls (GAPDH) and presented relative to the control group. Values are presented as mean ± SEM of five independent observations. The significance is shown as * P < 0.05 compared to the control group.

In the eCG-primed female mouse, E_2 _treatment did not cause a significant decrease in the expression of mPRβ mRNA at two hours, similarly to its effects in the immature mouse model. In contrast to the immature model, P_4 _treatment significantly down-regulated expression of mPRβ at this time (two hours). The mPRγ expression was also significantly reduced by P_4 _(six hours), whereas no effects where observed with the injections of E_2_, ICI or CDB (Fig. 4).

### Effects of gonadotropins on mPRβ and mPRγ expression in mouse fallopian tube

**Figure 5:**
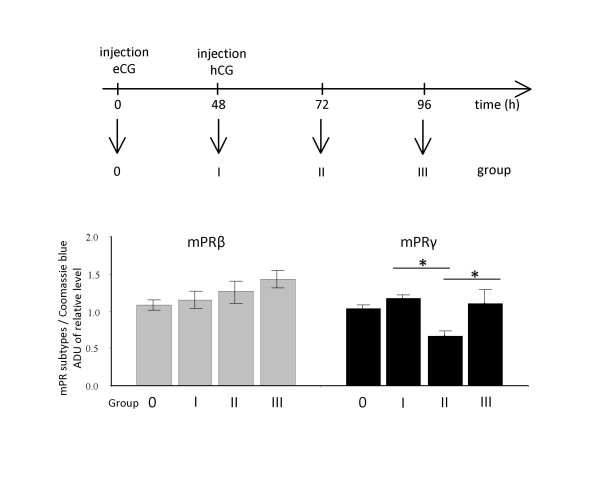
**Expression of mPRβ and γ in fallopian tubes of mice induced to ovulate by injections of eCG (0 h) and hCG (48 h)**. Mice were sampled at times 0, 48, 72 and 96 hours post eCG-injection and analyzed by Western blotting. Samples from ten mice were pooled and the values are presented as mean ± SEM of three independent pools for each group. All groups were compared with each other, applying Bonferroni's post-hoc test. The statistical significance is shown as * P < 0.05.

Treatment of immature mice with eCG did not affect the expression of the mPR proteins 48 h post injection. An additional injection with hCG transiently reduced the expression of mPRγ protein after 24 h, whereas the expression of mPRβ remained unaffected (Fig. 5).

### Expression of mPRβ and mPRγ in fallopian tubes from cycling women

**Figure 6:**
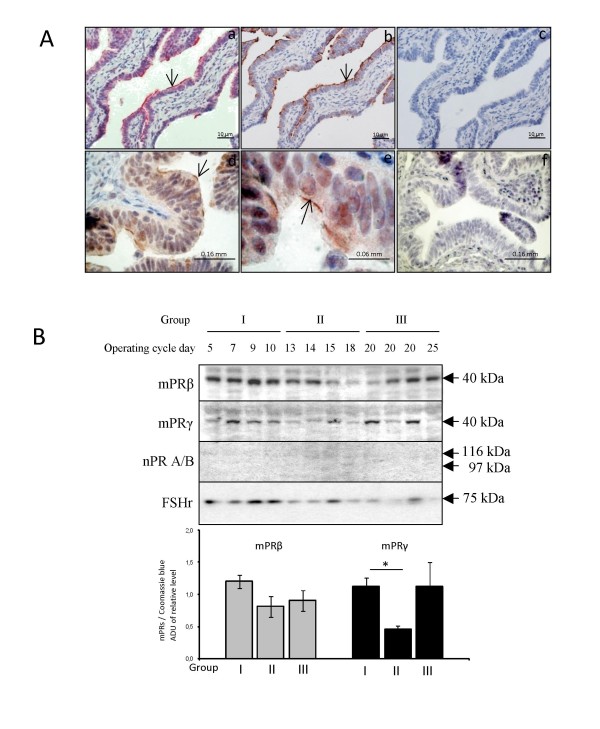
**Localization (A) and regulation (B) of mPRβ and γ in the human fallopian tube during the normal reproductive cycle**. The expression of mPRβ (**A, a**), β-tubulin (**A, b**) and mPRγ (**A, d-e**) are visualized by immunohistochemistry using specific antibodies. Subpanels **c **and **f **are control sections where the primary antibody was omitted. Another membrane bound protein, the FSH receptor (FSH) and the nuclear progesterone receptors were also analyzed to confirm that the sample preparation indeed contained membrane proteins. Sampled were obtained from patients in the follicular (group I), periovulatory (group II) and the luteal (group III) phase of the menstrual cycle. Protein levels of the mPRs are presented in the bar graph as arbitrary densiometric values normalized to whole protein in Coomassie blue staining. Values are presented as mean ± SEM of four independent observations. For mPRγ, assumptions for the general linear model was not met despite transformation of data (unequal variances), thus three independent t-tests (allowing unequal variances) were applied to compare stages I, II and III followed by a Bonferroni correction for multiple comparisons. The statistical significance is shown as * P < 0.05.

Similar to the localization in mice, human mPRβ was found in the distal part of the cilia of epithelial cells (Fig. 6A; a, b), whereas mPRγ was associated with the apical membrane of ciliated epithelial cells (Fig. 6A; d, e). Western blotting of human fallopian tubes also demonstrated expression of both mPRβ and mPRγ proteins with molecular weights of about 40 kDa (Fig. 6B). The expression of mPRγ in women sampled around ovulation was significantly lower than the expression in preovulatory women (Fig. 6B).

## Discussion

This study demonstrates a specific expression of mPRβ and mPRγ proteins in ciliary cells of both mouse and human fallopian tube epithelia. In contrast to earlier reports [43], we show a distinct expression of mPRβ on the cilia proper and of mPRγ at the base of the same cilia. We hypothesize that mPRβ and mPRγ have cooperative, but still distinctive functions in controlling ciliary movement and, thus, gamete transport in the fallopian tube of mammals. A demonstrated down-regulation of both mPRs after exogenous P_4 _treatment adds support to the previously proposed roles of the mPRs as functional P_4 _receptors [12,29]

We previously reported a detailed tissue distribution of mPRγ in male and female mice [20]. In the present study, we show that the mPRβ protein is expressed in reproductive tissues such as fallopian tube, ovary and testis of both male and female mice. In other species, including human, pig, rat and fish, the expression of mPRβ has been reported not only in reproductive tissues, but in non-reproductive tissues as well, including brain, heart and liver [29,31,34,36,38,40,44]. This was also the case in the mouse; however, the relative expression level of mPRβ between different organs appears to vary between species. The mPRβ appears to be expressed more evenly between tissues compared with mPRγ. In contrast to the high expression level of mPRγ in liver, mPRβ was almost undetectable in this organ. There were no marked differences in expression between the sexes for mPRβ, except for the reproductive tissues. We did not find a particularly high level of mPRβ expression in the brain, as previously reported [29,34]; however, this may be because we only studied the general tissue distribution in pre-pubertal animals.

Membrane progesterone receptor β was found in the motile cilia of epithelial cells in fallopian tube of both mice and women. The co-localization of mPRβ and β-tubulin IV, a cellular marker for cilia, also confirms the specific localization. In contrast, Romero-Sánchez et al. (2008) reported immunostaining of all epithelial cells, including both secretory and ciliated cells, in the human fallopian tube using the same mPRβ antibody as in our study. However, the localization within these cell types was unclear [43]. We have experienced that different fixation and staining techniques can give rise to a rather general intracellular staining using this and other antibodies (unpublished data). Qui et al. (2008) also show that the staining technique can be crucial, as addition of Triton X100 switched the staining by another antibody against mPRβ from the plasma membrane to a general intracellular staining in porcine cumulus cells [40]. Therefore, it is important to optimize the protocol to achieve the most specific staining possible. Several other studies, which used the same antibody used in the present study, report a plasma membrane localization of mPRβ in different cells and species, including human myocytes [33], human T lymphocytes and Jurkat cells [51] and mouse gonadotropes [52]. Other studies report an intracellular localization of mPRβ and γ, possibly in the endoplasmic reticulum [35,40,43,53]. The transcript for mPRβ has endoplasmatic reticulum export and endocytosis internalization motifs, which is in agreement with a potential intracellular localization [40,54]. It is important to stress that steroids easily pass the plasma membrane, thus the ligand(s) for mPRβ could quite possibly reach the receptor irrespective of its cellular localization.

Within the fallopian tube of mice and women, both mPRβ and γ are expressed exclusively in the ciliary epithelial cells. We previously reported that mPRγ was associated with the apical membrane at the base of the cilia of these cells [20]. Here, we show that mPRβ is expressed on the same cells, but in contrast to mPRγ, the receptors are found on the cilia themselves. The close association of both receptors with the cilia suggests an involvement in the regulation of ciliary activity by P_4_. In the cow, P_4 _decreases ciliary beat frequency within 15 minutes, and the effect cannot be blocked by a nuclear P_4 _receptor antagonist [9]. The co-occurrence of both receptors in the same cells also provides a possibility for a cooperative role. Cooperative roles for different mPR subtypes have been demonstrated previously in the induction of oocyte maturation in fish and in the regulation of PGR transactivation in human myometrial cells [33,55]. Interestingly, recent studies indicate the stimulatory effects of progestins on flagella activity and motility of fish and human sperm are mediated through a mPR subtype, mPRα [56,57]. A clear relationship between the abundance of the mPR protein on sperm membranes and sperm motility has been observed both in fish and humans [56,57]. Taken together the results suggest a possible widespread involvement of mPRs as intermediaries in progesterone modulation of the activities of ciliated and flagellated cells.

The classical PGR is also expressed in the fallopian tube; however, there is somewhat conflicting data regarding its distribution [45,46,58]. In the mouse, immunostaining for PGR was reported in the nuclei of luminal epithelial, stroma and smooth muscle cells [46,58], whereas in women the staining for PGR was restricted to the nuclei of epithelial and stromal cells [59]. In another study, no immunostaining was found for the classical PGR in the nuclei of any cell type in the mouse fallopian tube, whereas an intense staining was demonstrated in cilia proper [45], quite similar to the staining for mPRβ reported here. In this context, it may be stressed that there are no apparent sequence similarities between the classical PGR and mPRβ, thus little risk for one antibody to have high affinity for the other receptor. Several studies report a possible non-genomic, rapid signaling of P_4 _through PGR in different cell types [23,33,60]. Also, a plasma membrane localization of the classical PGR has also been suggested [14,61,62]. Together, this suggests that the ciliated cells of the fallopian tubes are equipped with a suite of P_4 _receptors, which all could be involved in controlling the beating of the cilia or possibly being involved in feedback systems by controlling receptor expression.

There is accumulating evidence that the family of mPRs (α, β and γ) act as receptors. Criteria, supporting a receptor function of the mPRs include information on plausible structure, tissue specificity, sub-cellular localization, steroid binding, signal transduction, hormonal regulation and biological relevance [12,29]. Although the aim of the present study has not been to scrutinize the classification of the mPRs as receptors, the generated data adds to the body of observations supporting such a role. One important observation is the plasma-membrane-association of mPRβ and mPRγ on the ciliary cells, i.e. a plausible sub-cellular localization. Also, the location of mPRβ on cilia of the fallopian tube, a cell-type known to respond rapidly within minutes to P_4_, is coherent with a receptor-role of mPRβ and mPRγ. Furthermore, treatment with exogenous P_4_down-regulates both mPRβ and mPRγ in gonadotropin-primed female mice within a few hours in the mouse fallopian tube. Indeed, it is very common that steroid receptors are regulated by their own ligand [29,30]. The functional fish progestin 17, 20β-dihydroxy-4-pregnen-3-one (17,20β-DHP) down-regulates mPRβ mRNA in the ovary of the channel catfish [63]. In human myometrial cells, however, P_4 _does not affect the expression of mPRβ [33]. We could only demonstrate a down-regulation by P_4 _in gonadotropin-primed mice, but not in immature mice. Thus, it appears that the regulation of mPRs by P_4 _differs between species, tissues and developmental stages.

The regulation of mPRβ and mPRγ by exogenous P_4 _could be mediated via a direct stimulation of P_4 _receptors on the cililary cells, or for example by modulating the release of gonadotropins. Receptors for FSH in the fallopian tube have been reported, but their physiological function in this organ is yet unknown [64]. In estrogen-primed, castrated rats P_4 _induces the release of both FSH and LH [65]. This model resembles the eCG-injected immature mouse model where the eCG injection induces endogenous estrogen production. We could demonstrate a regulation of both mPRβ and mPRγ by P_4 _in mice primed with eCG, but not in immature mice. This observation would be coherent with P_4 _regulating the mPRs via a hypothalamic/pituitary action and a stimulated gonadotropin secretion, although this hypothesis needs to be further tested. There was a similar trend of a reduced expression of both mPRβ and mPRγ in the estrogen-treated eCG-primed mice, consistent with an expected feedback on the gonadotropin release, but the effect was not statistically significant. On the other hand, E_2 _rapidly and significantly reduced the expression of mPRβ, but not mPRγ in the immature mouse model, clearly suggesting a gonadotropin-independent regulation. Tentative E_2 _and P_4 _response elements have been identified upstream from both the mPRβ and mPRγ genes [34], suggesting the possibility of a direct effect mediated via the classical steroid receptors. Direct regulation in vitro of mPRs by P_4 _and other progestin hormones has also been demonstrated previously in human myocytes and teleost ovaries [29,33]. As the travelling cumulus complex actively produces and releases steroids [15-18], the epithelium of the fallopian tube probably encounters higher steroid levels for a brief time period than do most cells in the body. Thus, it is possible that it is not the circulating steroids that are most important for regulating mPR expression in the fallopian tube, but rather steroids produced inside the fallopian tube. Indeed, the activation of sperm swimming by P_4 _is thought to be due to a gradient of locally produced P_4 _as the spermatozoa approaches the ovum inside the fallopian tube [66]. Notably, there is no intra-tubal steroid production in either the immature or the eCG model.

Access to samples from women undergoing tubal ligations at different time points in their menstrual cycles allowed us to start addressing the physiological regulation of mPRs in the human fallopian tube. Both mPRβ and mPRγ are expressed on the ciliated cells of the human fallopian tube, similar to their location in mice. Interestingly, the expression of mPRγ was lower in women sampled close to mid cycle, i.e., around the time of ovulation, whereas there was no clear regulation of mPRβ. The general pattern of a down-regulation of mPRγ and a more stable expression of mPRβ was also observed in mice induced to ovulate via two injections with gonadotropins. This pattern suggests that there are commonalities between the regulation of mPRs in the fallopian tube of mice and women, adding confidence to the use of mice as a human model in this context. The lack of apparent regulation of mPRβ in both ovulating mice and women may be somewhat difficult to interpret in relation to the results from the steroid injections, as there indeed are marked variations in the circulating steroid levels before and after ovulation in both species. Possibly, more frequent sampling could have revealed variations in the receptor expression.

## Conclusion

In conclusion, we have demonstrated the presence of mPRβ and γ in ciliated cells of the fallopian tube of mice and women and the regulation of these receptors by P_4 _and/or E_2_. These observations further strengthen the role of the ciliated cells of the fallopian tube as important physiological targets for P_4_. Taken together, our results support the hypothesis of an involvement of the mPRs in P_4_-regulated gamete transport in mammals.

## Competing interests

The authors declare that they have no competing interests.

## Authors' contributions

MN and DGJL conceived and designed the study, interpreted the results and drafted the manuscript. MN performed the sampling of the mice, carried out the RNA and protein preparations and conducted the Western blot analysis. BW performed the Taqman analysis and immunohistochemistry analysis. PT provided the specific antibody against mPRβ used in this study and gave suggestions and inputs on the drafts of the manuscript. ATK provided the clinical specimens. HB participated in the design of the study and the interpretation of the results and commented on the draft manuscript. All authors read and approved the final manuscript.
